# NY-ESO-1 Based Immunotherapy of Cancer: Current Perspectives

**DOI:** 10.3389/fimmu.2018.00947

**Published:** 2018-05-01

**Authors:** Remy Thomas, Ghaneya Al-Khadairi, Jessica Roelands, Wouter Hendrickx, Said Dermime, Davide Bedognetti, Julie Decock

**Affiliations:** ^1^Cancer Research Center, Qatar Biomedical Research Institute, Qatar Foundation, Hamad Bin Khalifa University, Doha, Qatar; ^2^Immunology, Inflammation, and Metabolism Department, Tumor Biology, Immunology, and Therapy Section, Division of Translational Medicine, Sidra Medicine, Doha, Qatar; ^3^Department of Surgery, Leiden University Medical Center, Leiden, Netherlands; ^4^Translational Cancer Research Facility, National Center for Cancer Care and Research, Doha, Qatar

**Keywords:** cancer-testis antigen, NY-ESO-1, adoptive T cell therapy, vaccine, immune checkpoint

## Abstract

NY-ESO-1 or New York esophageal squamous cell carcinoma 1 is a well-known cancer-testis antigen (CTAs) with re-expression in numerous cancer types. Its ability to elicit spontaneous humoral and cellular immune responses, together with its restricted expression pattern, have rendered it a good candidate target for cancer immunotherapy. In this review, we provide background information on NY-ESO-1 expression and function in normal and cancerous tissues. Furthermore, NY-ESO-1-specific immune responses have been observed in various cancer types; however, their utility as biomarkers are not well determined. Finally, we describe the immune-based therapeutic options targeting NY-ESO-1 that are currently in clinical trial. We will highlight the recent advancements made in NY-ESO-1 cancer vaccines, adoptive T cell therapy, and combinatorial treatment with checkpoint inhibitors and will discuss the current trends for future NY-ESO-1 based immunotherapy. Cancer treatment has been revolutionized over the last few decades with immunotherapy emerging at the forefront. Immune-based interventions have shown promising results, providing a new treatment avenue for durable clinical responses in various cancer types. The majority of successful immunotherapy studies have been reported in liquid cancers, whereas these approaches have met many challenges in solid cancers. Effective immunotherapy in solid cancers is hampered by the complex, dynamic tumor microenvironment that modulates the extent and phenotype of the antitumor immune response. Furthermore, many solid tumor-associated antigens are not private but can be found in normal somatic tissues, resulting in minor to detrimental off-target toxicities. Therefore, there is an ongoing effort to identify tumor-specific antigens to target using various immune-based modalities. CTAs are considered good candidate targets for immunotherapy as they are characterized by a restricted expression in normal somatic tissues concomitant with a re-expression in solid epithelial cancers. Moreover, several CTAs have been found to induce a spontaneous immune response, NY-ESO-1 being the most immunogenic among the family members. Hence, this review will focus on NY-ESO-1 and discuss the past and current NY-ESO-1 targeted immunotherapeutic strategies.

## Cancer-Testis Antigens (CTA)

Cancer-testis antigens form a family of antigens that are encoded by 276 genes, comprising more than 70 gene families (1). Approximately 50% of all CTA genes form multigene families on the X chromosome and are referred to as CT-X genes (2). These CTAs are located in specific clusters along the chromosome with the highest density in the Xq24–q28 region (3). CTA expression is largely restricted to testicular germ cells and placenta trophoblasts with no or low expression in normal adult somatic cells (3, 4).

Interestingly, several CTAs have been found to be re-expressed in a variety of epithelial cancers. For example, using the RNA sequencing dataset from the Genotype–Tissue Expression project, Rooney et al. identified 60 CTAs with absent expression in normal tissues and ectopic expression in numerous tumor types, including melanoma, head and neck, lung, liver, stomach, and ovarian cancer (5). Across cancer types, tumors can be classified as CTA-rich, intermediate, or poor based on the frequency of CTA expression. CTA-rich tumors include melanoma, ovarian, lung, and bladder cancer; the group of CTA-moderate tumors comprises breast, bladder, and prostate cancer, while renal cell carcinoma, colorectal cancer, and lymphoma/leukemia are categorized as CTA-poor tumors (4, 6). Several reports suggest that there might be a preference for specific CTA re-expression in certain tumor types, exemplified by MAGEA1–4 in 70% of metastatic melanomas, ACRBP in 70% of ovarian tumors and NY-ESO-1 in 46% of breast tumors (7). CTA expression patterns have also been associated with disease stage. For example, whereas no expression of the SSX family of CTAs could be observed in benign prostate tissue, 23% of metastatic prostate cancer lesions showed re-expression of SSX proteins (8). Although CTA expression may be increased in bulk primary tumors, this might not be reflected on the single cell level. For instance, microdissection of ovarian cancer specimens demonstrated considerable intra-tumoral heterogeneity of NY-ESO-1 expression (9). Heterogenous expression can also be found across metastatic foci originating from one primary lesion (10). Intra-tumoral heterogeneity could partly explain the different extent to which certain CTAs are re-expressed in tumors. In addition, discrepancies between RNA and protein expression levels are not uncommon and may contribute to the variety of expression levels reported. There is a great need for studies with direct comparison of RNA and protein expression levels in the same samples, which is impeded by the lack of specific commercially available CTA antibodies.

The mechanisms underlying the aberrant re-expression of CTAs include DNA demethylation and histone posttranslational modification with recent evidence also supporting a role for miRNA-mediated regulation (2, 11). Interestingly, demethylating agents such as 5-aza-2-deoxycytidine are capable to induce re-expression of certain CTAs specifically in tumor cells but not in normal epithelial cells (12–16).

Cancer-testis antigens are not only re-expressed in tumor tissues but they are also believed to be immunogenic proteins as many members of the family have been shown to elicit spontaneous cellular and humoral immune responses in cancer patients. The first CTA identified, MAGE-A1, was discovered through its ability to induce an autologous cytotoxic T lymphocyte response in a melanoma patient (17). Since then, several other CTAs have been identified as immunogenic tumor-associated antigens (TAAs) including SSX-2, NY-ESO-1, and various members of the BAGE, GAGE, and MAGE families (2, 18).

Despite the increasing amount of data on CTA expression in normal and neoplastic tissues, their functions remain largely elusive. Transgenic mouse models have revealed several CTA members to play a key role in male fertility (7). A handful of CTAs have been implicated in cell metabolism, cytoskeleton dynamics, double-strand break repair, maintenance of genomic integrity, and regulation of mRNA expression during spermatogenesis. Although increasing number of studies are demonstrating CTA re-expression in cancer, their functional role in oncogenesis is largely unexplored. More recent data points to a role for CTAs in the regulation of epithelial-to-mesenchymal transition as well as tumor cell survival (19).

Given their largely restricted expression in adult somatic tissues and their immunogenic potential, CTAs have been considered good candidate targets for cancer immunotherapy. To date, NY-ESO-1 is the most promising CTA for immune-based interventions as its tumor expression is clearly correlated with the induction of an immune response in a wide range of malignancies (2). Therefore, this review will focus on NY-ESO-1 in relation to its expression pattern and biological function; and will also discuss some of the past and recent developments in NY-ESO-1 tumor immunology and immunotherapy. This review does not aim to cover all available literature on NY-ESO-1 in cancer as this has become a very large area of research. Therefore, certain studies might have been missed in this review for which we apologize in advance.

## NY-ESO-1 Expression in Normal Tissues

The CTA New York Esophageal Squamous Cell Carcinoma-1 (NY-ESO-1) is also known as cancer-testis antigen 1B (CTAG1B) and is encoded by the gene *CTGAG1B* which is located on the Xq28 region of the X chromosome.

NY-ESO-1 is an archetypical example of a CTA with restricted expression to germ cells and placental cells and re-expression in tumor cells. During embryonic development, NY-ESO-1 expression can be detected as early as 13 weeks and is the highest at 22–24 weeks (20). While NY-ESO-1 expression is maintained in the spermatogonia and primary spermatocytes, its expression quickly decreases in the female oogonia (3, 20–22). RNA, but not protein, expression of NY-ESO-1 has been detected at low levels in ovarian and endometrial tissue, albeit its biological relevance is as yet unclear (21, 23).

NY-ESO-1 is an 18-kDa protein with 180 amino acids featuring a glycine-rich N-terminal region and a strongly hydrophobic C-terminal region with a Pcc-1 domain. The NY-ESO-1 protein has been shown to be homologous to two other CTA located in the same region; LAGE-1 and ESO3 (24). While NY-ESO-1 and LAGE-1 encode very homologous proteins with restricted expression in adult testis, the ESO3 protein has a rather low similarity with both and has been reported to be expressed in various somatic tissues (25). Screening of cDNA expression libraries for T cell epitope discovery has revealed that NY-ESO-1 can be generated from an alternative open reading frame, resulting in a 58 amino acid-long protein that is recognized by tumor-reactive T cells (26).

Although very little is known about the biological function of NY-ESO-1, possible functions can be deducted from its structure and expression pattern. The presence of the conserved Pcc-1 domain suggests that it might be involved in cell cycle progression and growth (27). Coexpression with melanoma antigen gene C1, a member of the MAGE family of CTAs (28), further supports involvement in cell cycle progression and apoptosis given the prominent role of MAGE proteins in these cellular processes (29). In addition, its restricted expression pattern suggests that it may have a role in germ cell self-renewal or differentiation. This notion is further supported by the nuclear localization of NY-ESO-1 in mesenchymal stem cells in contrast to the predominant cytoplasmic expression in cancer cells. Upon differentiation of mesenchymal stem cells, NY-ESO-1 expression is downregulated which suggests a possible role in cell proliferation of stem cells and cancer cells (30). Furthermore, NY-ESO-1 expression is increased in glioma cancer stem cells compared to differentiated cells, concurrent with histone acetylation and DNA hypomethylation (31).

## NY-ESO-1 Expression in Cancer

### Tumor Expression

NY-ESO-1 expression has been reported in a wide range of tumor types, including neuroblastoma, myeloma, metastatic melanoma, synovial sarcoma, bladder cancer, esophageal cancer, hepatocellular cancer, head and neck cancer, non-small cell lung cancer, ovarian cancer, prostate cancer, and breast cancer (21, 32–46). Within these tumor types, the expression frequency of NY-ESO-1 differs greatly with the most commonly expressing tumors being myxoid and round cell liposarcoma (89–100%), neuroblastoma (82%), synovial sarcoma (80%), melanoma (46%), and ovarian cancer (43%) (37, 47–51). Other tumor types show protein expression of NY-ESO-1 in the range of 20–40%. In addition, numerous studies have reported RNA expression data which might differ considerably from protein expression levels as determined by immunohistochemistry; and only few studies have investigated both RNA and protein expression.

Important to note is that the majority of cancer types show a heterogeneous expression of NY-ESO-1, which could limit the treatment response to NY-ESO-1, targeted treatment. The most homogenous expression has been reported in myxoid and round cell liposarcomas (94%) and synovial sarcomas (70%) which might be related to the promising results that have been obtained in adoptive cellular immunotherapy trials (47, 49).

### Humoral and Cellular Immune Response

The first evidence of a spontaneous immune response against NY-ESO-1 was reported in an esophageal cancer patient (52). Using the tumor mRNA of one patient with a squamous cell carcinoma of the esophagus, the authors constructed a tumor cDNA expression library that was immunoscreened with the serum of the same patient. Using the SEREX technology (*Se*rological analysis of *re*combinant cDNA *ex*pression libraries), NY-ESO-1 was identified as a tumor antigen that could elicit high-titer IgG humoral responses.

Humoral immune responses against NY-ESO-1 can be detected in a variety of cancer patients, including patients with colorectal, lung, breast, prostate, gastric, and hepatocellular cancer (53–61). As most of these studies focus on one cancer type, the study of Oshima et al. is worth noting as they performed a large serological study on 1,969 specimens from patients with various cancer types (62). The authors could detect serum NY-ESO-1 autoantibodies across all cancers, with the highest frequency in esophageal cancer (32%), followed by lung cancer (13%), hepatocellular cancer (11%), prostate and gastric cancer (10%), colorectal cancer (8%), and breast cancer (7%). Analysis of healthy individuals revealed no spontaneous NY-ESO-1 humoral response.

NY-ESO-1 has also been shown to induce a cellular immune response. The first report of a simultaneous humoral and cellular response against NY-ESO-1 was observed in a metastatic melanoma patient with a high-titer antibody response (63). Three HLA-A2 restricted epitopes in NY-ESO-1 were identified as the recognition sites for CD8+ cytotoxic T lymphocytes. In a follow-up study, the same team validated their findings by detection of NY-ESO-1-specific CD8+ T cells in 10 out of 11 melanoma patients who carry NY-ESO-1 antibodies (64). In a later study, they also identified three HLA-DRB4*0101–0103, MHC class II epitopes that were recognized by CD4+ T lymphocytes from two melanoma patients (65). In addition, others identified HLA-DRB1*0401 and HLA-DP4 restricted epitopes in the carboxy-terminal end of NY-ESO-1 which can be recognized by CD4+ T lymphocytes from melanoma patients (66–69). The peptide containing the HLA-DP4-restricted epitope could also generate HLA-A2-restricted CD8+T cells, suggesting that the peptide could be used as a cancer vaccine to induce both CD4+ and CD8+ T cell responses (70). This opens up the number of immunotherapeutic approaches that can be employed against NY-ESO-1-positive tumors inducing the humoral immune system as well as the cellular CD4+ and CD8+ T cell compartments, either individually or in combination. Interestingly, in healthy individuals NY-ESO-1 specific CD4+ T-cell precursors were found to be actively suppressed by regulatory T cells, suggesting that the cytokine milieu of the tumor microenvironment can dictate and impede natural NY-ESO-1 antitumor immune responses (71, 72). This notion is of great importance and should be taken into account during the design of novel immune-based interventions.

### NY-ESO-1 as Biomarker

NY-ESO-1 expression has been found across tumor types to correlate with several characteristics of advanced disease, including higher differentiation grade, lymph node metastasis, and clinical stage (23). The value of NY-ESO-1 expression as a prognostic biomarker remains controversial. While its expression has been linked to a poor clinical outcome in some cancers, no correlation or conflicting results have been found in others. For instance, in non-small cell lung cancer, hepatocellular carcinoma, head and neck cancer, gastrointestinal cancer, multiple myeloma, and malignant melanomas NY-ESO-1 tumor expression has been associated with a higher risk of recurrence, poor treatment response and shorter survival (73–83). In contrast, in non-Hodgkin’s lymphoma NY-ESO-1 tumor expression was associated with early stage disease (84). Early reports on ovarian and breast cancer did not show any significant correlation with prognosis (37, 85, 86). Immune responses to NY-ESO-1 have also been investigated in the context of circulating biomarkers, providing a non-invasive way for monitoring disease progression and treatment response. The extent of a NY-ESO-1-specific humoral immune response has been found to increase with disease progression and to decrease with disease regression (32, 33, 38, 60, 62, 87–89). A comprehensive overview of NY-ESO-1 expression and immunogenicity in different cancers has been provided by Esfandiary and Ghafouri-Fard (22). After the publication of that review, new findings showed that the detection rate of NY-ESO-1 antibodies in esophageal cancer gradually increases with disease stage, going from 16% in stage I to 42% in stage IV (62). Further, in colorectal cancer, the presence of NY-ESO-1 antibodies has been recently correlated with several prognostic clinicopathological parameters including depth of tumor invasion, clinical stage, lymph node, and distant metastasis (89). Changes in NY-ESO-1 antibodies over time can be indicative of disease regression and could be used as markers for disease monitoring as demonstrated in 12 patients with different tumor types (88). In four out of five patients (two bladder cancer, two melanoma, one non-small cell lung cancer) with a decrease in NY-ESO-1 humoral response, a reduction in tumor burden and/or metastases was observed. Likewise, NY-ESO-1-T-cell responses have been investigated as prognostic markers. In metastatic melanoma, the presence of circulating NY-ESO-1-specific T lymphocytes has been associated with better prognosis, improving overall survival from 6 to 21 months (90).

## NY-ESO-1 Directed Immunotherapy

**Figure 1:**
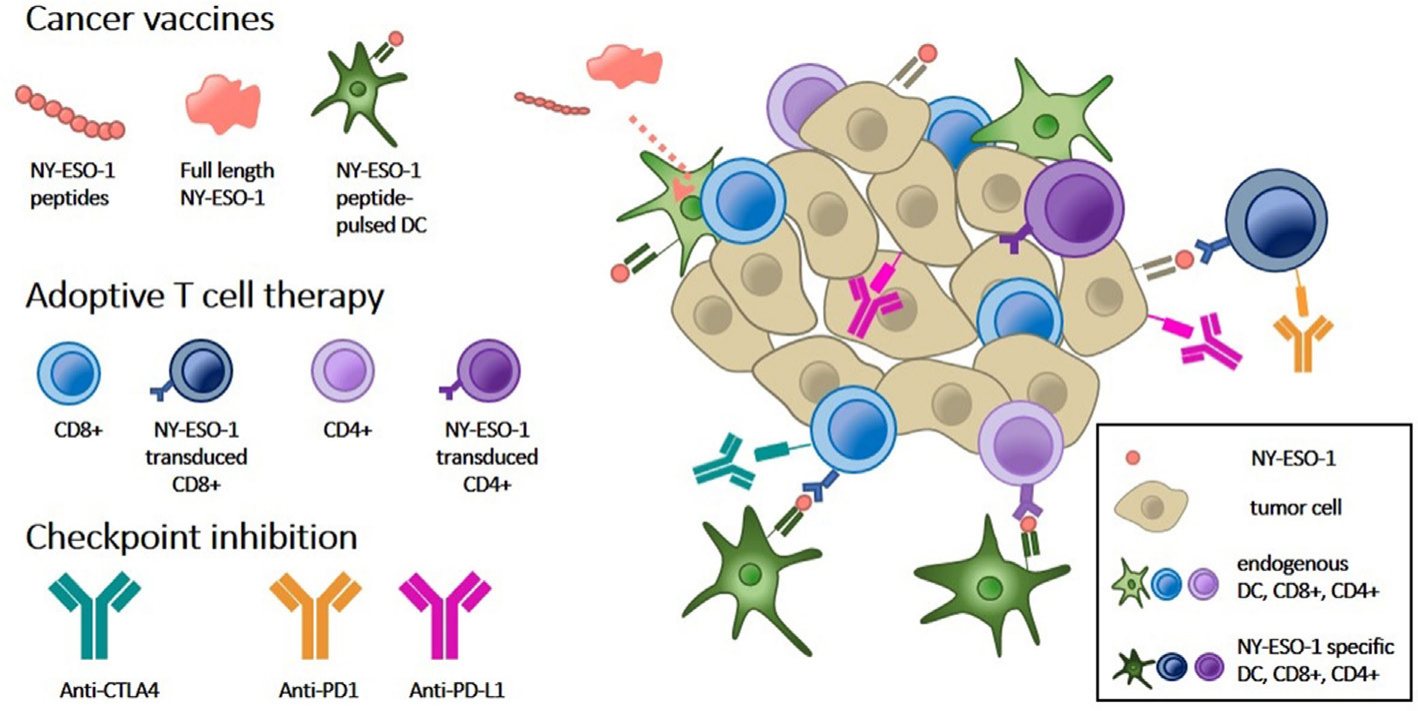
Overview of NY-ESO-1 targeted approaches for cancer immunotherapy. Immune-mediated tumor rejection can be induced by targeting tumor-specific NY-ESO-1 expression by the following three approaches: (1) cancer vaccines consisting of NY-ESO-1-derived peptides or full-length protein are taken up by dendritic cells (DCs, light green) and subsequently presented to the tumor immune microenvironment. Vaccination of DCs pulsed with NY-ESO-1 peptides (dark green) directly stimulates T cells. (2) Adoptive T cell therapy by transduction of CD8+ or CD4+ T cells with NY-ESO-1 T cell receptors (dark blue, dark purple, respectively) directs them against NY-ESO-1, presented by tumor cells (cream). (3) Both types of immunotherapy can be combined with immune checkpoint inhibitors which block inhibitory signals between DCs and T cells (anti-CTLA-4) and between T cells and tumor cells (anti-PD1 and anti-PD-L1).

NY-ESO-1 is widely believed to be a good candidate target for immunotherapy and some promising results have been obtained in early phase I/II studies. Its restricted expression in normal tissues in combination with its widespread expression across tumor types renders NY-ESO-1 a target with limited off-target toxicities and broad applications in numerous cancer types. Furthermore, its strong immunogenic nature suggests that there is an opportunity to boost the natural immune response against this TAA. To date, there are 12 active, 31 recruiting, and 5 proposed clinical trials targeting NY-ESO-1 using various immune-based interventions (http://www.clinicaltrials.gov). In this review, we will discuss the ongoing clinical trials, summarized in Tables 1–3, and highlight some of the completed trials using a NY-ESO-1 vaccination approach, adoptive cellular therapy, or combination treatment with immune checkpoint inhibitors (Figure 1).

**Table 1:** NY-ESO-1 cancer vaccines currently in clinical trial.

NCT number	Other IDs	Interventions	Conditions	Status
NCT01967758	13-012A	NY-ESO-1 bacterial vaccine ADU-623	Astrocytic tumors|glioblastoma multiforme|anaplastic astrocytoma|brain tumor	Active, not recruiting
NCT01536054	I 199911|NCI-2011-02964	ALVAC(2)-NY-ESO-1 (M)/TRICOM vaccine|mTOR inhibitor (Sirolimus)	Stage II–IV and recurrent fallopian tube cancer/ovarian epithelial cancer/primary peritoneal cavity cancer	Active, not recruiting
NCT02833506	I 277115|NCI-2016-00811|P30CA016056|R01CA158318	Recombinant NY-ESO-1 Protein vaccine|mTOR inhibitor (Sirolimus)	Stage II–IV and recurrent fallopian tube cancer/ovarian epithelial cancer/primary peritoneal cavity cancer	Not yet recruiting
NCT02224599	Kirovax 003|BSK01 DC vaccine	Peptide-pulsed DC vaccine	Advanced solid tumors	Recruiting
NCT02692976	EudraCT 2012-002531-29	Multi peptide (NY-ESO-1, MUC1) -pulsed myeloid and plasmacytoid DC vaccine	Prostate cancer	Active, not recruiting
NCT01883518	MC-01-2013|ADCVCTAST	Allogeneic tumor lysate (NY-ESO-1, MAGE-A3) -pulsed DC vaccine	Soft tissue sarcoma	Recruiting
NCT02334735	GCO 14-0780	Multi peptide (NY-ESO-1 and Melan-A/MART-1) -pulsed DC vaccine	Melanoma	Recruiting
NCT02122861	ID-LV305-2013-001	DC lentiviral vector vaccine (LV305)	Melanoma/non-small cell lung cancer/sarcoma	Active, not recruiting
NCT02387125	IMDZ-C131	CMB305 (peptide-pulsed DC vaccine LV305 +G305 recombinant NY-ESO-1 protein vaccine)|TLR4 agonist (G100)	Sarcoma|melanoma|non-small cell lung cancer|ovarian cancer	Recruiting
NCT02129075	NCI-2014-00898|CITN-07-FLT3L|U01CA154967	DEC-205/NY-ESO-1 Fusion Protein vaccine (CDX-1401)|Recombinant Flt3 Ligand (CDX-301)	Stage II–IV melanoma	Active, not recruiting
NCT02166905	I 248613|NCI-2014-00771|P30CA016056	DEC-205/NY-ESO-1 Fusion Protein (CDX-1401)|IDO1 inhibitor (Epacadostat)	Fallopian tube carcinoma|ovarian carcinoma|primary peritoneal carcinoma	Recruiting
NCT02750995	AZACTA	Multi peptide vaccine (NY-ESO-1, PRAME, MAGE-A3, WT-1)|demethylating agent Decitabine	Myelodysplastic syndrome|acute myeloid leukemia	Recruiting

*CMB305, peptide-pulsed DC vaccine LV305 + G305 recombinant NY-ESO-1 protein vaccine; DC, dendritic cell; CDX-1401, DEC-205/NY-ESO-1 Fusion Protein vaccine*.

**Table 2:** NY-ESO-1 adoptive T cell therapy modalities currently in clinical trial.

NCT number	Other IDs	Interventions	Conditions	Status
NCT02366546	1301-01	TBI-1301	Advanced solid tumors	Recruiting
NCT02869217	1301-02	TBI-1301	Advanced solid tumors	Recruiting
NCT03250325	1301-03	TBI-1301	Synovial sarcoma	Recruiting
NCT03047811	NY-TCR WXH 2016	NY-ESO-1 TCR-T cells	Advanced solid tumors	Recruiting
NCT02457650	201504002	NY-ESO-1 TCR-T cells	Metastatic solid tumors	Recruiting
NCT01795976	ATTACK-OG|12_DOG14_22	NY-ESO-1 TCR-T cells	Esophageal cancer	Active, not recruiting
NCT03093350	H-39209 TACTIC	Multi TAA T cells (NY-ESO-1, MAGEA4, PRAME, survivin and SSX2)	Breast cancer	Not yet recruiting
NCT03192462	H-40378 TACTOPS	Multi TAA T cells (NY-ESO-1, MAGEA4, PRAME, survivin and SSX2)	Pancreatic cancer	Not yet recruiting
NCT02239861	H-35425, TACTASOM	Multi TAA T cells (NY-ESO-1, MAGEA4, PRAME, survivin and SSX2)	Rhabdomyosarcoma	Recruiting
NCT02291848	H-35626, TACTAM	Multi TAA T cells (NY-ESO-1, MAGEA4, PRAME, survivin and SSX2)	Multiple myeloma	Recruiting
NCT02494167	H-36346 ADSPAM	Multi TAA T cells (WT1, NY-ESO-1, PRAME, and survivin)	Acute myeloid leukemia, myelodysplastic syndrome	Recruiting
NCT03175705	Beijing Youan Ethics[2017]06	Multi TAA T cells (GPC-3, NY-ESO-1, AFP)	Hepatocellular carcinoma	Recruiting
NCT02774291	2015-5254|NCI-2015-01781|P30CA013330	Murine NY-ESO-1 TCR-T cells	Metastatic solid tumors	Not yet recruiting
NCT01967823	130214|13-C-0214	Murine NY-ESO-1 TCR-T cells	Metastatic solid tumors	Recruiting
NCT01567891	ADP-0011-001|230612	NY-ESO-1^c259^-T cells	Ovarian cancer	Recruiting
NCT01350401	ADP 01611	NY-ESO-1^c259^-T cells	Metastatic melanoma	Active, not recruiting
NCT01343043	ADP 04511	NY-ESO-1^c259^-T cells	Synovial sarcoma	Recruiting
NCT02992743	ADP-0011-007	NY-ESO-1^c259^-T cells	Advanced myxoid, round cell liposarcoma	Recruiting
NCT01892293	ADP-0011-002	NY-ESO-1^c259^-T cells	Multiple myeloma	Active, not recruiting
NCT02588612	ADP-0011-004	NY-ESO-1^c259^-T cells	Non-small-cell lung cancer	Recruiting
NCT01352286	ADP 01411	NY-ESO-1^c259^-T cells|stem cell transplantation	Multiple myeloma	Active, not recruiting
NCT03029273	2016-63	TAEST16001	Recurrent non-small cell lung cancer	Recruiting
NCT03159585	TS20161229	TAEST16001	solid tumors	Recruiting

*TAA, tumor-associated antigen; TAEST16001, TCR affinity enhancing specific T cell therapy with autologous T cells transduced with affinity-enhanced NY-ESO-1 TCR; TBI-1301, NY-ESO-1 specific TCR-transduced T cells; TCR, T cell receptor*.

**Table 3:** NY-ESO-1 combinatorial immune-based interventions currently in clinical trial.

NCT number	Other IDs	Interventions	Conditions	Status
NCT01946373	MAT-02|2012-000450-63	Peptide-pulsed DC vaccine|TILs	Melanoma	Recruiting
NCT01176474	MCC-15651|NCI-8316	NY-ESO-1_157–165_/gp100_280–288_ vaccine|PD-1 inhibitor (Nivolumab)|PD-1 inhibitor (Nivolumab) + CTLA-4 inhibitor (Ipilimumab)	Stage III–IV melanoma	Active, not recruiting
NCT01176461	MCC-15400|NCI-P-7997|CA209-006/007|10-15526-99-01	Multi peptide vaccine (MART-1, NY-ESO-1, gp100_209–217_, gp100_280–288_|PD-1 inhibitor) (Nivolumab)	Melanoma	Active, not recruiting
NCT02609984	IMDZ-C232	CMB305|PD-L1 inhibitor (Atezolizumab)	Sarcoma	Active, not recruiting
NCT03206047	NCI-2017-01030|I 285416|10017|P30CA016056	DEC-205/NY-ESO-1 fusion protein vaccine (CDX-1401)|demethylating agent|PD-L1 inhibitor (atezolizumab)	Recurrent fallopian tube carcinoma|ovarian carcinoma|primary peritoneal carcinoma	Recruiting
NCT03017131	i 283616|NCI-2016-01477|P30CA016056|P50CA159981	NY-ESO-1 TCR-T cells|demethylating agent	Recurrent fallopian tube carcinoma|ovarian carcinoma|primary peritoneal carcinoma	Not yet recruiting
NCT01333046	H-27471-TACTAL|TACTAL	Multi TAA T cells (NY-ESO-1, MAGEA4, PRAME, survivin and SSX2)|decitabine	Hodgkin lymphoma|non-Hodgkin lymphoma|Hodgkin disease	Recruiting
NCT01697527	12-000153|NCI-2012-01548	NY-ESO-1 TCR-T cells|NY-ESO-1_157–165_ peptide-pulsed DC vaccine	Advanced solid tumors	Recruiting
NCT03240861	15-000511|NCI-2017-00896|Ribas NYESO SCT Cancer|P30CA016042	NY-ESO-1 TCR-transduced peripheral blood mononuclear cells and peripheral blood stem cells	Advanced solid tumors	Recruiting
NCT02650986	I 258514|NCI-2015-02080|P30CA016056	NY-ESO-1 TCR-T cells|TGFbDNRII-transduced TILs	Advanced solid tumors	Recruiting
NCT03168438	ADP-0011-008|KEYNOTE-487	NY-ESO-1_c259_-T cells|PD-1 inhibitor (pembrolizumab)	Multiple myeloma	Recruiting
NCT02775292	NYM|15-001433|NCI-2016-00201|Ribas NYESO + Nivolumab Cancer|P30CA016042	NY-ESO-1 TCR-T cells|NY-ESO-1_157–165_ -pulsed DC vaccine|PD-1 inhibitor (nivolumab)	Advanced solid tumors	Recruiting
NCT02070406	13-001624|NCI-2014-00221|P30CA016042	NY-ESO-1 TCR-T cells|NY-ESO-1_157–165_ pulsed DC vaccine|CTLA-4 inhibitor (ipilimumab)	Advanced solid tumors	Recruiting

*CMB305, peptide-pulsed DC vaccine LV305 + G305 recombinant NY-ESO-1 protein vaccine; CDX-1401, DEC-205/NY-ESO-1 Fusion Protein vaccine; DC, dendritic cell; TCR, T cell receptor; TAA, tumor-associated antigen; TILs, tumor-infiltrating lymphocytes; PD-L1, programmed death ligand 1; CTLA-4, cytotoxic T-lymphocyte-associated protein 4; PD-1, programmed cell death protein 1*.

### NY-ESO-1 Cancer Vaccines

The current NY-ESO-1 cancer vaccine trials have evolved considerably since the first clinical trials that were conducted more than one decade ago. Many advances have been made in peptide discovery and vaccine formulation. We now have an armatorium of individual synthetic peptides, individual and complexed recombinant proteins, as well as a variety of adjuvant formulations to our disposal. Various adjuvants have been rigorously tested for their ability to enhance cytotoxic CD8+ T lymphocyte activity in response to exposure to MHC class I-restricted peptides. These adjuvants vary from granulocyte/macrophage colony-stimulating factor to montanide-ISA-51 (Montanide), polyinosinic–polycytidylic acid-stabilized by lysine and carboxymethyl cellulose (Poly-ICLC), incomplete Freund’s adjuvant, saponin-based adjuvant (ISCOMATRIX), cholesteryl pullulan, and monophosphoryl lipid A (91, 92). A combination of various factors has been proposed as novel adjuvant (APH); consisting of alum, polysaccharides and the short synthetic innate defense-regulator peptide HH2 (93). NY-ESO-1 vaccination with this adjuvant significantly increased humoral and cellular responses and reduced the melanoma burden in mice. Another strategy to enhance vaccination efficacy is to induce CD4+ immune responses to support the priming and maintenance of CD8+ cytotoxic T lymphocytes (92). To date, 21 distinct epitopes restricted to at least five different HLA-class II alleles have been identified in NY-ESO-1. The peptides NY-ESO-1_80–109_ and NY-ESO-1_157–165_, associated with, respectively, CD4+ and CD8+ T cell responses, have been shown to be the most immunogenic. Using full-length recombinant protein can further enhance the extent of the induced immune responses as both class I and class II epitopes are available for antigen presentation and processing. For instance, vaccination of melanoma patients with recombinant full-length NY-ESO-1 alone or in combination with the ISCOMATRIX adjuvant resulted in a strong induction of NY-ESO-1 specific antibodies, as well as an increase in specific CD4+ and CD8+ T cells (94). Using longer peptides also has the potential to induce a stronger immune response. For example, vaccination with the 20-mer NY-ESO-1_91–110_ peptide, covering multiple epitopes, induced both humoral and cellular CD4+ and CD8+ T cell responses. Furthermore, stable disease was achieved in 3 out of 10 cancer patients (95). Another approach to enhance NY-ESO-1 vaccination responses is the use of a prime and boost schedule, consisting of an initial vaccination, the prime, with 1 cancer vaccine followed by administration of a second vaccine, the boost. Using a recombinant NY-ESO-1 vaccine/recombinant fowlpox-NY-ESO-1 vaccine prime-boost regimen, both humoral and cellular responses against a broad range of NY-ESO-1 epitopes have been observed (96, 97). Other modifications to the acellular vaccine approach currently in clinical trial are the use of a bacterial vector for NY-ESO-1 vaccine delivery (NCT01967758, Table 1); and combinatorial treatment of NY-ESO-1 vaccination with the mTOR inhibitor Sirolimus (NCT01536054, NCT02833506, Table 1) or with the demethylating agent Decitabine (NCT02750995, Table 1).

Since dendritic cells (DCs) are the dominant antigen-presenting cells and are strong activators of T cells, numerous studies have investigated the use of peptide-pulsed DCs as cellular vaccines (98). Further advances in the field have demonstrated an added benefit of including toll-like receptor (TLR) agonists as an adjuvant. The rationale behind this lies in the TLR-mediated activation of DCs, and the induction of T helper 1-cell responses (99). Indeed, combination treatment with a TLR3 (Hiltonol), TLR7 (imiquimod), TLR7/8 (resiquimod), or TLR9 (CpG 7909) agonist has been shown to enhance humoral and cellular responses in a significant proportion of cancer patients (100–102). The safety and efficacy of NY-ESO-1-pulsed DCs is currently under study in various clinical trials (Table 1), either alone (NCT02692976, NCT01883518, NCT02334735, NCT02224599) or in combination with a NY-ESO-1 protein vaccine and TLR4 agonist (NCT02387125). Specific targeting of the DCs can also be achieved using the DEC-205/NY-ESO-1 fusion protein (CDX-1401), which targets the NY-ESO-1 protein for DC endocytosis through the DEC-205 receptor. This fusion protein is currently in clinical trial in combination with the recombinant Flt3 ligand CDX-301 to promote DC development (NCT02129075, Table 1), or with the IDO1 inhibitor Epacadostat (NCT02166905, Table 1), or with infusion of tumor-infiltrating lymphocytes (TILs) (NCT01946373, Table 3). Cancer vaccines can also be targeted toward DCs through TLRs. One example of this approach is the use of a NY-ESO-1 encoding LV305 lentivirus, targeting DCs through TLR3 and TLR7, which induced a strong cellular immune response with significant disease regression in one patient with metastatic, treatment-refractory synovial sarcoma (103). This case report is part of an ongoing phase I clinical trial (NCT02122861, Table 1) investigating the use of intradermal NY-ESO-1-specific lentiviral DC-targeting in melanoma, non-small cell lung cancer, ovarian cancer, and sarcoma. A second example of acellular dendritic-based therapy is the use of Fc receptor-mediated uptake of liposome-encapsulated adjuvants and/or drugs with subsequent DC activation (104). Combining a NY-ESO-1 protein vaccine with the liposome-encapsulated chemotherapeutic drug doxorubicin and the demethylation agent Decitabine enhanced the specific humoral and CD8+ immune responses in 67 and 50% of patients with relapsed epithelial ovarian cancer (105). Stable disease was obtained in 50% of patients (5/10) with a median duration of 6.3 months, and one patient had a partial response (10%) with a duration of 5.8 months.

### NY-ESO-1 Adoptive T Cell Therapy

NY-ESO-1 cancer vaccines have proven to elicit both humoral and cellular responses; however, few complete responses have been obtained using this approach. Therefore, the focus for immune-based intervention against NY-ESO-1 has largely changed over time toward the development of genetically engineered T lymphocytes. Based on the knowledge gained from vaccine studies, T cells directed against specific NY-ESO-1 epitopes have been engineered and tested for their ability to eradicate tumors. Adoptive T cell therapy with HLA-A2 restricted NY-ESO-1/LAGE-1 transduced CD8+ T cells has improved the clinical response rates and overall survival of treatment-refractory melanoma and synovial cell sarcoma patients. In a first cohort, approximately half of patients with metastatic melanoma or synovial cell sarcoma who received NY-ESO-1 transduced CD8+ T cells and IL-2 showed a clinical response (106). 2 out of 11 metastatic melanoma patients exhibited a complete response and 1 patient had a partial response. Four out of six patients with synovial cell sarcoma experienced partial responses, representing the first evidence of successful NY-ESO-1 adoptive T cell therapy in non-melanoma patients. In a follow-up study, the authors expanded their cohort with 9 melanoma and 12 synovial cell sarcoma patients and provided an update on the clinical responses of the first cohort (107). Combining both cohorts, objective responses were obtained in 61% of patients with synovial cell sarcoma with 5-year overall survival rates of 14%, and in 55% of melanoma patients with overall 5-year survival rates of 33%. Furthermore, the same adoptive T cell treatment resulted in near complete or complete response in 80% of multiple myeloma patients with a median progression-free survival of 19.1 months (108). Numerous clinical trials are currently investigating the safety and efficacy of NY-ESO-1 transduced CD8+ T cells, using NY-ESO-1 either as a single target or as part of a multi-TAA target. While some current trials, summarized in Table 2, study NY-ESO-1 specific or multi-TAA TCR-transduced T cells in a range of advanced solid tumors (NCT03047811, NCT02457650, NCT02869217, NCT02366546), others focus on subgroups of patients with esophageal cancer (NCT01795976), breast cancer (NCT03093350), pancreatic cancer (NCT03192462), rhabdomyosarcoma (NCT02239861), hepatocellular carcinoma (NCT03175705), synovial sarcoma (NCT03250325), or hematological cancers (NCT02494167, NCT02291848). NY-ESO-1-specific murine TCR-transduced T cells in combination with high-dose IL-2 and chemotherapy are currently under evaluation in metastatic cancer patients (NCT02774291, NCT01967823, Table 2). In addition, there are two phase I/II clinical trials investigating the safety and activity of T cell transduction with an affinity-enhanced T cell receptor (TCR) for NY-ESO-1 and LAGE-1 (NY-ESO-1^c259^ T cells) in patients with relapsed or advanced multiple myeloma (NCT01892293, NCT01352286, Table 2). Similarly, adoptive transfer of affinity-enhanced NY-ESO-1 transduced T cells is under evaluation (Table 2) in metastatic melanoma (NCT01350401), ovarian cancer (NCT01567891), synovial sarcoma (NCT01343043), myxoid/round cell liposarcoma (NCT02992743) and non-small cell lung cancer (NCT03029273, NCT02588612), and in a patient cohort with a variety of advanced solid cancers (NCT03159585). Furthermore, the safety, feasibility, and efficacy of NY-ESO-1 or multi TAA transduced T cell therapy in combination with other modalities is currently under review, including combination with treatment with the demethylating agent Decitabine (NCT03017131, NCT01333046, Table 3), NY-ESO-1_157–165_-pulsed DCs (NCT01697527, Table 3), transduced peripheral blood stem cells (NCT03240861, Table 3), or dominant-negative transforming growth factor-beta receptor II transduced TILs (NCT02650986, Table 3).

Although the majority of adoptive T cell therapy studies focus on cytotoxic CD8+ T lymphocytes, re-educated CD4+ T cells also show potential to eradicate cancer cells. For example, a case study demonstrated that treatment with HLA-DP4-restricted NY-ESO-1 transduced CD4+ T cells can induce complete regression of a refractory metastatic melanoma, with a durable response ongoing at 22 months (109). However, since most cancer cells do not express MHC class II molecules the efficacy of CD4+ based immunotherapy *in se* is rather limited. Presentation of the HLA-DP restricted NY-ESO-1_157–170_ epitope was enhanced *in vitro* by directing the antigen to the macro-autophagy pathway using a fusion protein of NY-ESO-1 and the autophagy molecule Atg8/LC3 (110). The authors also reported that intercellular transfer of NY-ESO-1 by endocytosis increased antigen presentation. Therefore, agents targeting NY-ESO-1 cellular release and/or macro-autophagy could provide a new avenue to improve CD4+ immunotherapy. The lack of MHC class II tumor expression can also be addressed by engineering CD4+ T cells that can recognize HLA-class I-restricted peptides. Using *in vitro* and *in vivo* mouse models, this approach was found to induce cancer cell cytotoxicity and cytokine production (111–117). A recent study by Tan et al. demonstrated that HLA-A2-restricted NY-ESO-1_157–165_ transduced CD4+ T cells displayed higher binding affinity for the peptide than CD8+ T cells and were able to induce cancer cell cytotoxicity (118). These preclinical findings suggest that HLA-class I-redirected CD4+ T cells could improve the antitumor response of current adoptive T cell therapies.

### Combination Treatment With Checkpoint Inhibitors

The immune response is naturally kept in check by immune checkpoint molecules [cytotoxic T-lymphocyte-associated protein 4 (CTLA-4), programmed cell death protein 1 (PD-1) and programmed death ligand 1 (PD-L1)] in order to prevent over-activation of the immune response resulting in autoimmune disease. While the PD-1 pathway inhibits T cell activation during the effector phase, the CTLA-4 pathway plays an important role in naïve T cell activation during the priming phase (119). Binding of PD-1 on T cells to its ligands, PD-L1 and programmed death ligand 2, inhibits T-cell proliferation and activation and reduces T cell survival (120). CTLA-4 is a CD28 homolog on the T-cell cell membrane that through competitive binding will inhibit the costimulatory signal of CD28:B7 binding, similarly resulting in reduced T cell proliferation, differentiation, and survival (121). CTLA-4 expression can also increase T-cell mobility, thereby reducing the contact time between antigen-presenting cells and T-cells and indirectly inhibiting T cell activation (122). In addition, CTLA-4 is constitutively expressed on regulatory T cells, which through competitive binding with B7 and/or induction of B7 internalization reduces the availability of B7 on antigen-presenting cells to form the CD28:B7 costimulatory signal for T cell activation and survival (123, 124). Cancer cells can hijack these T-cell regulatory pathways to dampen the antitumor response. One approach in cancer immunotherapy is to unleash the natural immune response by inhibiting these checkpoint molecules using specific inhibitors. Patients who do not respond to immune checkpoint blockade may still benefit from combinatorial treatment with cancer antigen-specific therapy. For instance, treatment with the CTLA-4 checkpoint inhibitor ipilimumab has been reported to induce specific NY-ESO-1 humoral and cellular immune responses in patients with ovarian cancer, prostate cancer, and metastatic melanoma (100, 125–135). Ipilimumab-treated melanoma patients with a NY-ESO-1-specific humoral response at baseline more often experienced an antitumor response and improved survival if accompanied by a NY-ESO-1 specific CD8+ cellular immune response (131).

In addition, NY-ESO-1-specific CD4+ T cells isolated from a metastatic melanoma patient after treatment with ipilimumab were able to directly lyse autologous cancer cells, suggesting an added clinical benefit of eliciting a NY-ESO-1 CD4+ cellular response (134). Together, these findings indicate that combination treatment of checkpoint inhibitors with NY-ESO-1 targeted treatment might result in enhanced and more durable clinical responses. However, this might not be true for all tumor types. Although NY-ESO-1 is highly expressed in sarcoma, antibodies against the antigen are not as common thereby questioning the added clinical benefit of NY-ESO-1 targeted treatment in combination with checkpoint blockade (136–138). Therefore, a pilot phase I trial has been designed to determine the safety and efficacy of combining CTLA-4 blockade with NY-ESO-1 adoptive T cell therapy and NY-ESO-1 vaccination in patients with locally advanced or metastatic malignancies (NCT02070406, Table 3).

Similarly to CTLA-4 inhibition, inhibition of the immune checkpoint molecule PD-1 induces a NY-ESO-1 specific CD8+ cytotoxic immune response (139). Further evidence supporting a potential added benefit from PD-1 inhibition to NY-ESO-1 targeted immunotherapy comes from NY-ESO-1_157–165_ peptide vaccination of melanoma patients, demonstrating an upregulation of the T cell inhibitory molecules PD-1, Tim-3 and BTLA in NY-ESO-1 CD8+ T cells (140–143). *In vitro* blockade of both PD-1 and Tim-3 increased cytotoxic cell proliferation and cytokine secretion of NY-ESO-1_157–165_ CD8+ T cells (140, 144). Similarly, tumor-derived NY-ESO-1_92–100_ CD8+ T cells isolated from ovarian cancer patients showed an upregulation of the inhibitory molecules PD-1 and LAG-3 with dual blockade enhancing the proliferation and cytokine production (145). Treatment of metastatic melanoma patients with the PD-1 inhibitor nivolumab in combination with the NY-ESO-1_157–165_ peptide vaccine revealed that the response rate in both ipilimumab-pretreated and -naïve patients was 25% (146). Serological analyses showed a correlation of high pretreatment NY-ESO-1 CD8+ T cells with disease progression, suggesting that these cells might express several inhibitory molecules, such as Tim-3 and LAG-3. In contrast, no difference in NY-ESO-1 cellular immune response has been observed between non-small cell lung cancer patients responding to PD-1 blockade by nivolumab and non-responders (147). The first experimental evidence supporting the use of PD-1 inhibitors together with NY-ESO-1 adoptive T cell therapy came from a preclinical study using a lung cancer xenograft mouse model (148). In this study, NY-ESO-1 transduced T cells could infiltrate tumors and reduce tumor growth by 50%; however, the cells could not reduce tumor burden and showed upregulation of PD-1, Tim-3, and LAG-3. PD-1 blockade in addition to injection of transduced T cells reduced the tumor burden with an additional 35%. Ongoing clinical trials are exploring the safety of combining NY-ESO-1 peptide vaccination with Nivolumab (NCT01176461, Table 3), or with both Nivolumab and Ipilimumab (NCT01176474, Table 3). Other trials are determining the safety and feasibility of combining NY-ESO-1 transduced T cell therapy with Nivolumab and NY-ESO-1 peptide-pulsed DC vaccine in advanced solid cancer (NCT02775292, Table 3), or with Pembrozilumab in multiple myeloma (NCT03168438, Table 3). Furthermore, targeting the ligand for PD-1, PD-L1, on tumor cells with Atezolizumab is currently under investigation in combination with NY-ESO-1 pulsed DC vaccination in patients with sarcoma (NCT02609984, Table 3), and in combination with the DEC-205/NY-ESO-1 fusion protein (CDX-1401) vaccine and the demethylation agent guadecitabine in patients with recurrent ovarian, fallopian tube, or primary peritoneal cancer (NCT03206047, Table 3).

## Where to Go Next?

NY-ESO-1 targeted treatment has come a long way, targeting the antigen using various approaches from peptide and protein vaccination to adoptive T cell therapy and combinational treatment modalities. Promising results have been obtained, driving new clinical trials in numerous solid cancers.

Nevertheless, a pressing concern for NY-ESO-1 based therapy is the considerable inter- and intra-heterogeneity of NY-ESO-1 tumor expression, which could significantly limit the extent of tumor cell eradication using NY-ESO-1 targeted treatment. Since the expression of many CTA, including NY-ESO-1, is regulated by methylation, one approach that is being pursued is to increase the tumoral re-expression of NY-ESO-1 by demethylating agents prior to NY-ESO-1 targeted treatment. An early study by Weiser et al. demonstrated that NY-ESO-1 expression could be induced *in vitro* by treatment with the DNA demethylating agent 5-Aza-2′-deoxycytidine, an effect which could be enhanced by sequential treatment with a deacetylase inhibitor (149). Since then, several preclinical studies have shown that demethylation not only increases expression of NY-ESO-1 specifically in tumor cells, but also induces specific CD8+ immune responses and tumor cell cytotoxicity; and when used in combination with NY-ESO-1 immunotherapy it reduced the tumor burden and prolonged the survival in several mouse models (150–155). These experimental findings suggest that epigenetic modulation may enhance or even enable NY-ESO-1 adoptive immunotherapy in poorly immunogenic tumor types.

On another note, even though promising results have been obtained with various NY-ESO-1 cancer vaccine approaches, some reservations need to be made. Since cancer vaccines are often based on synthetic peptides, the question arises whether the induced immune response reflects or complements the natural immune response against endogenous antigen expression. Naturally induced CD8+ immune responses against NY-ESO-1 are commonly directed against an HLA-A2-restricted epitope within the amino acid region 157–165 or 157–167 (156). Comparison of naturally and vaccine-induced CD8+ responses revealed that these cells exhibit structurally conserved but distinct TCR features (157). These findings suggest that synthetic peptides used for vaccination may not accurately reflect the naturally processed antigen and antitumor immune response.

Another important factor to take into account is the impact of the microenvironment on the outcome of immune-modulating treatments. The cancer-immunity cycle is a well-known concept and has become the framework for immunotherapy research. The cancer-immunity cycle describes the various steps that have to be completed to obtain successful eradication of tumor cells, including release of cancer cell antigens, cancer antigen presentation, priming and activation of T cells, trafficking of T cells to tumors, infiltration of T cells into tumors, recognition of cancer cells by T cells, and finally killing of cancer cells (158). Therefore, the make of the inflammatory milieu and the presence of immune suppressive cells can have a profound effect on treatment efficacy. Tumor cells are capable of escaping the antitumor immune response by hindering each of the steps of the cancer-immunity cycle. First, tumors can escape immune surveillance by altering the expression of tumor antigens. This has raised the concern that the presence of spontaneous tumor antigen-immune responses might induce epitope spreading as a result of prolonged immune pressure. For instance, interim analysis of a phase II study (NCT02609984, Table 3) using a combinatorial approach of PD-L1 inhibition and NY-ESO-1 DC-targeting revealed specific humoral and cellular responses in 50% of patients with synovial sarcoma and myxoid round cell liposarcoma but also antigen epitope spreading in 20% of patients (159). Second, the presentation of tumor antigens can also be altered as has been demonstrated in inflammatory melanoma (160). The difference in activity between the non-inflammatory proteasome and the immunoproteasome has been shown to result into a dissimilar repertoire of epitopes that impedes the ability of T cells to recognize and target the tumor cells.

Third, the presence or induction of immunosuppressive cells can have a profound effect on the treatment outcome. In advanced melanoma it was shown that a single NY-ESO-1 epitope could induce CD4+ T cell responses as well as stimulate T regulatory cells (161). Further investigation revealed that these specific T regulatory cells are derived from CD4+ CD25− T cells. Hence, inhibition of the peripheral conversion of CD4+ CD25− T cells into specific T regulatory cells may improve treatment outcome. Further, treatment with the NY-ESO-1/ISCOMATRIX vaccine induced NY-ESO-1 specific T regulatory responses, most commonly recognizing the HLA-DP4-restricted NY-ESO-1_157–170_ peptide (162). In tumor tissue, T regulatory cells with specificity toward the HLA-DR-restricted NY-ESO-1_115–132_ peptide could be observed. Together, these findings suggest that chronic antigen exposure can result in the suppression of both the circulating and local antitumor immune response through the stimulation and induction of antigen-specific T regulatory T cells. Similarly, chronic hepatitis B infection has been shown to increase the numbers of specific T regulatory cells in the peripheral blood and liver of patients with hepatocellular carcinoma (163). *In vitro* investigation of co-culture of peripheral blood mononuclear cells with HBV-transfected hepatoma cell lines revealed an increase in T regulatory cells together with an upregulation of FoxP3 and the immune checkpoint CTLA-4. Interestingly, these T regulatory cells were capable of suppressing not only HBV-induced but also NY-ESO-1 tumor antigen-induced immune responses.

## Concluding Remarks

Since its discovery, NY-ESO-1 has been investigated as an anti-cancer target for immune-based interventions. Several approaches have been explored *in vitro, in vivo*, and in clinical trials. The vast majority of clinical trials focus on solid cancers in the advanced stage. Currently, there are 12 clinical trials registered using a NY-ESO-1 cancer vaccine, 23 using modified T cells, and 13 using combinatorial immunotherapy. As the field of immunotherapy is evolving, limitations to these approaches are becoming apparent which can be tackled by refining the current methods or addressing them from a different angle as discussed in this review. Exploring such new strategies have resulted in several novel treatments that are currently in clinical trial.

## Author Contributions

JD conceived, designed, and drafted the manuscript. RT and GA-K wrote sections of the manuscript. JR designed the figure and critically revised the manuscript. WH, DB, and SD critically revised the manuscript and contributed to writing the final manuscript. All authors read and approved the manuscript for publication.

## Conflict of Interest Statement

The authors declare that the research was conducted in the absence of any commercial or financial relationships that could be construed as a potential conflict of interest.
